# Improving primary health care delivery in Bihar, India: Learning from piloting and statewide scale-up of *Ananya*

**DOI:** 10.7189/jogh.10.021001

**Published:** 2020-12

**Authors:** Gary L Darmstadt, Kevin T Pepper, Victoria C Ward, Sridhar Srikantiah, Tanmay Mahapatra, Usha Kiran Tarigopula, Debarshi Bhattacharya, Laili Irani, Janine Schooley, Indrajit Chaudhuri, Priyanka Dutt, Padmapriya Sastry, Radharani Mitra, Sara Chamberlain, Sophia Monaghan, Priya Nanda, Yamini Atmavilas, Niranjan Saggurti, Evan Borkum, Anu Rangarajan, Kala M Mehta, Safa Abdalla, Jess Wilhelm, Yingjie Weng, Suzan L Carmichael, Hina Raheel, Jason Bentley, Wolfgang A Munar, Andreea Creanga, Shamik Trehan, Dilys Walker, Hemant Shah

**Affiliations:** 1 Department of Pediatrics, Stanford University School of Medicine, Stanford, California, USA; 2 CARE India, Patna, India; 3 Bill and Melinda Gates Foundation, Delhi, India; 4 Population Council, New Delhi, India; 5 Project Concern International, Delhi, India, and San Diego, California, USA; 6 BBC Media Action (India), New Delhi, India; 7 Mathematica, Princeton, New Jersey, USA; 8 Department of Epidemiology and Biostatistics, University of California San Francisco, San Francisco, CA, USA; 9 Johns Hopkins Bloomberg School of Public Health, Baltimore, MD, USA; 10 Quantitaitve Sciences Unit, Department of Medicine, Stanford University, Stanford, CA, USA; 11 George Washington University Milken Institute School of Public Health, Washington DC, USA; 12 Dr Reddy’s Foundation, Hyderabad, India; 13 Department of Obstetrics and Gynecology and Reproductive Services, University of California San Francisco, San Francisco, California, USA

## Abstract

In 2010, the Bill and Melinda Gates Foundation (BMGF) partnered with the Government of Bihar (GoB), India to launch the *Ananya* program to improve reproductive, maternal, newborn and child health and nutrition (RMNCHN) outcomes. The program sought to address supply- and demand-side barriers to the adoption, coverage, quality, equity and health impact of select RMNCHN interventions. Approaches included strengthening frontline worker service delivery; social and behavior change communications; layering of health, nutrition and sanitation into women’s self-help groups (SHGs); and quality improvement in maternal and newborn care at primary health care facilities. *Ananya* program interventions were piloted in approximately 28 million population in eight innovation districts from 2011-2013, and then beginning in 2014, were scaled up by the GoB across the rest of the state’s population of 104 million. A Bihar Technical Support Program provided techno-managerial support to governmental Health as well as Integrated Child Development Services, and the JEEViKA Technical Support Program supported health layering and scale-up of the GoB’s SHG program. The level of support at the block level during statewide scale-up in 2014 onwards was approximately one-fourth that provided in the pilot phase of *Ananya* in 2011-2013. This paper – the first manuscript in an 11-manuscript and 2-viewpoint collection on Learning from *Ananya: *Lessons for primary health care performance improvement – seeks to provide a broad description of *Ananya* and subsequent statewide adaptation and scale-up, and capture the background and context, key objectives, interventions, delivery approaches and evaluation methods of this expansive program. Subsequent papers in this collection focus on specific intervention delivery platforms. For the analyses in this series, Stanford University held key informant interviews and worked with the technical support and evaluation grantees of the *Ananya* program, as well as leadership from the India Country Office of the BMGF, to analyse and synthesise data from multiple sources. Capturing lessons from the *Ananya* pilot program and statewide scale-up will assist program managers and policymakers to more effectively design and implement RMNCHN programs at scale through technical assistance to governments.

High-performing primary health care (PHC) systems are essential for attainment of the Sustainable Development Goals (SDGs) and universal health coverage [1,2]. Several studies – while citing methodological limitations around large-scale studies of PHC initiatives – have captured consistent evidence in multiple low- and middle-income countries (LMICs) that investments in PHC systems can lead to improved health outcomes including increased access to essential health services, reduced child mortality, and reduced wealth-based disparities in mortality [1,3]. Further, there is a robust body of evidence correlating interventions delivered through specific service delivery channels or platforms with improved reproductive, maternal, newborn and child health and nutrition (RMNCHN) outcomes, including 1) strengthening the capabilities of frontline workers (FLWs) [4-7]; 2) skill development and staff training at public health facilities [8]; and, 3) exposure to multifaceted social and behaviour change communication (SBCC) programs, community participation efforts, and women’s self-help groups (SHGs), among other channels for individual and collective behaviour change [9-17]. Nevertheless, gaps in knowledge persist regarding how PHC systems in LMICs can acquire and sustain the organisational capacities and human resources required to consistently deliver high-quality services in ways that contribute to sustained and equitable improvements in health outcomes at the population level. Multiple studies have also pointed to significant gaps in PHC performance including major constraints in access to quality care, retention of a competent workforce, and advancement of patient-centeredness in care [18,19]. Effective scale-up of evidence-based health interventions across populations has been particularly challenging.****Key barriers to sustainable impact at scale include insufficient leadership and management capacity across multiple levels, especially at the top; the complexity of implementation across heterogeneous delivery platforms; insufficient development of methodological approaches for monitoring and evaluation (including measures and metrics) that address the complexity inherent to the operation of health systems and services in real-life scenarios; diminished implementation fidelity wherein interventions may be effective during piloting but falter under different conditions or reduced intensity at scale; and lack of engagement of local implementers and communities [20].

Designing a roadmap for making smart investments in PHC systems has been obfuscated by a lack of evaluation methods that can yield insights into what, how, for whom, and at what levels of the system interventions work. One review of 106 health system evaluations noted that very few adopted a comprehensive approach to assessing system-wide effects, and most evaluations only targeted one “building block” of the health system [21]. Moreover, only 32% and 5% of 76 evaluations reviewed in one study used quasi-experimental and experimental designs, respectively [1]. Even when evidence is generated that might be useful to policymakers and program designers, it can take many years for research to be widely disseminated, and even longer to become standard practice, while alternative interventions or approaches might continue to be deployed and to evolve further within the program or system where they originated.

In recent years, there has been a concerted effort to coalesce around the most effective PHC practices and measurement approaches through initiatives such as the Primary Health Care Performance Initiative [22] and *The Lancet* Global Health Commission on High Quality Health Systems in the SDG Era [23], as well as through the identification of critical research gaps [24,25]. There is a need to identify research methods which can provide policy-relevant learning, including policy and implementation research which seeks to provide generalisable evidence on what works in a variety of contexts and at scale [26].

*Ananya *(Hindi for “unique”), a large-scale RMNCHN technical support program funded by the Bill & Melinda Gates Foundation (BMGF) was implemented in the state of Bihar, India, beginning in late 2011. *Ananya* has important contributions to make to PHC systems research. The Bihar program provides an opportunity to learn how to effectively study a large-scale PHC program and its attempts to address key evidence gaps and yield new insights on improving PHC system performance, strengthen community-based interventions such as SBCC and SHGs, and enhance service delivery across populations. In this series, we share learnings from this technical assistance program beginning with deep implementation support to the Government of Bihar (GoB) in eight pilot districts (which was branded as *Ananya*) and subsequent adaptation and scale-up of implementation technical assistance under the Bihar Technical Support Program (BTSP) at significantly reduced intensity of support to the GoB across the 38 districts in Bihar. Our aim is to help inform the efforts of policymakers, system administrators and practitioners to more effectively achieve widespread RMNCHN impact at scale. This first paper provides an overview of the context of the *Ananya* program, as well as the program and evaluation designs. Subsequent papers in this series delve into greater detail on the multiple delivery platforms of the program and their impacts, including FLW capacity-building, SHGs, SBCC efforts, and quality improvement of facility-based care.

## Study setting

In 2010 when *Ananya* began, Bihar had recently emerged from decades of political upheaval and widespread breakdown of the rule of the law. The health sector suffered from pervasive and deeply entrenched deficiencies of leadership, funding, infrastructure, commodities, management, and staffing, and amongst the population, awareness and adoption of critical RMNCHN behaviours were low [27]. Bihar was performing well below India state averages across several surveys of critical RMNCHN indicators (Table S1 A-C in the **Online Supplementary Document)**. [28]. Given the large population and poor health indicators of Bihar, it was clear that improvements in the state’s health system could have a significant impact on progress toward national and global health goals.

Despite its challenges, Bihar was experiencing impressive economic growth as it emerged from prolonged stagnation, and the government was attempting to rapidly modernise its administration, infrastructure, and health services. High-level political committment to improvements in health and human development coincided with increased funds from the central government through the creation of the National Rural Health Mission (NRHM) in 2005, the Ministry of Health and Family Welfare (MoHFW)’s flagship program [29]. The NRHM was focused on RMNCH, with the goal of reaching the most marginalised communities with priority health interventions with flexible funding and space for state-led innovations.. The state’s health expenditure, spurred by NRHM funding, was growing rapidly [30]. A substantial contribution of the NRHM was the number of health workers, particularly District and Block Program Managers, Auxiliary Nurse Midwives (ANMs) and Accredited Social Health Activists (ASHAs) that were being contracted but often not fully trained and prepared to perform their roles [31]. The *Janani Bal Suraksha Yojana* program − a safe motherhood initiative under the NRHM − provided a conditional cash transfer to women for delivering in a health facility which catalysed unprecedented levels of institutional deliveries [32]. JEEViKA was launched in 2007 by the Bihar Rural Livelihoods Promotion Society under the Department of Rural Development, with the support of the World Bank Group [33]. The program focused on organising women in poor rural households through SHGs and related community institutions, helping them improve their livelihoods and enhance household incomes.

Concurrently, other development partners and donors were making significant investments in Bihar’s health system. UNICEF and the World Health Organisation had a preexisting presence in Bihar. The United Kingdom’s Department for International Development (DFID) had just launched their five-year $220 million program – Sector-Wide Approach to Strengthening Health in Bihar (SWASTH) – to strengthen health, nutrition, water and sanitation systems across Bihar [34]. The Norway India Partnership Initiative (NIPI) had also started to engage with the state government to support newborn care. Against this backdrop of mounting government and donor commitments to Bihar’s health system, the BMGF began to commit significant resources to Bihar, as complementary to other partner investments, but with a sharp program.

## *ANANYA* PROGRAM DESIGN AND IMPLEMENTATION

### Pilot phase (2010-2013)

**Figure 1:**
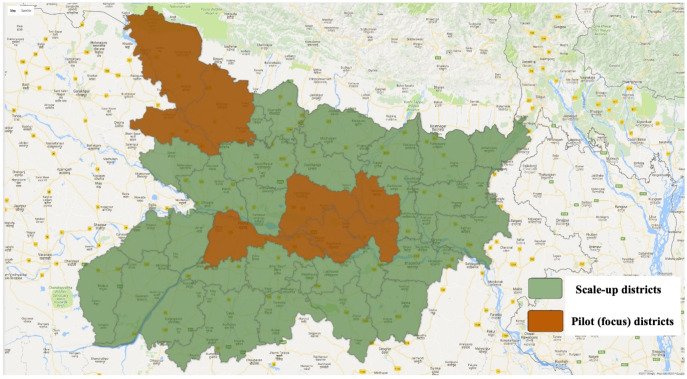
Location of the *Ananya* program in eight pilot (focus) districts (2012-2013), with subsequent expansion of the program statewide (2014-2017) to all 38 districts.

In 2010, BMGF formed a partnership with the GoB aimed to provide them with technical support, including the design and introduction of new solutions to long-standing barriers to improving coverage of RMNCHN interventions, for the purpose of accelerating progress towards the state’s ambitious health goals (Table S2 in the **Online Supplementary Document**). The program was designed at the outset with a built-in, five-year exit strategy for building technical capacity and transferring innovative strategies and tools to the government. Given the presence of the SWASTH program, as well as the strategic approach of the BMGF at that time, a decision was made not to adopt a health system strengthening approach in the beginning but rather to evaluate how best to implement evidence-based RMNCHN interventions at scale through home- and community-based behaviour change, and at the first level clinics and primary health centers which provide Basic Emergency Obstetric and Newborn Care (BEmONC). Thus, in the first two years of implementation (2011-2013), the *Ananya* program would focus on testing a range of innovations designed by *Ananya* partners for implementation across multiple public delivery platforms by GoB functionaries to increase the coverage of critical RMNCHN interventions in eight out of Bihar’s 38 districts (**Figure 1**), and then support the government with technical assistance as they scaled up successful solutions across the state. The focus districts in the pilot phase were located in two clusters in Bihar: one in the northwest region of the state (including East Champaran, West Champaran, and Gopalganj) and the other which was relatively accessible in the central region near the capital city of Patna (Patna, Samastipur, Begusarai, Saharsa, and Khagaria).

#### Theory of change

**Figure 2:**
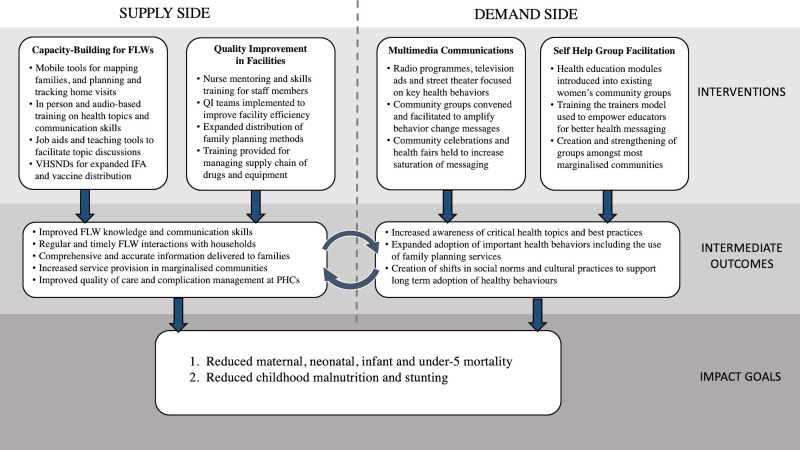
Initial *Ananya* Theory of Change. FLW – frontline worker, IFA – iron-folic acid, PHC – Primary Health Centre, QI – quality improvement, VHSND – Village Health Sanitation and Nutrition Day.

At the time of *Ananya*’s design, the GoB was seeking to address high rates of maternal, neonatal and child mortality, stunting and fertility. The GoB’s programs and thus the focus of *Ananya* was on supporting GoB implementation of a comprehensive set of evidence-based RMNCHN interventions and delivery platforms that targeted the critical 1000-day period between the start of a mother’s pregnancy and a child’s second birthday [35,36]. *Ananya* was aimed to make the GoB’s programs more effective in achieving goals chosen by the government (state and center), and involved no direct transfer of cash or material from BMGF or partners to the government. Rather, *Ananya* engaged partners in Bihar to work together with concerned governmental departments and programs in improving implementation design, prioritising interventions for achieving stated goals, and measuring and monitoring progress using indicators and credible sources of data that were otherwise unavailable to the government. The *Ananya* program’s initial “Theory of Change” (**Figure 2**) focused on supporting the GoB to: 1) Increase the quantity, timeliness, equity and quality of FLW interactions with households and communities; 2) Generate community demand and improve RMNCHN behaviours and health care-seeking practices, and 3) Improve the quality of childbirth, immediate postpartum and family planning services at PHC facilities.

#### *Ananya* program design

The program included multiple grants by BMGF, principally: 1) the Integrated Family Health Initiative (IFHI) led by CARE (Cooperative for Assistance and Relief Everywhere) India; 2) the Shaping Demand and Practices (SDP) grant led by BBC Media Action; 3) the *Parivartan* project led by Project Concern International (PCI) to layer health, nutrition and sanitation interventions within SHGs, coupled with a grant to Population Council to evaluate the impact of the *Parivartan* program; and 4) a grant to Mathematica to evaluate the impact of IFHI and SDP in focus districts during the pilot phase. Additional investments not covered in this paper collection included grants intended to: 1) Consolidate and standardise primary treatment for childhood illnesses including diarrhea, pneumonia and tuberculosis through a network of private rural medical practitioners [37]; 2) Improve quality of intrapartum care in private nursing homes; 3) Support the GoB to reach and sustain elimination of visceral leishmaniasis; 4) Improve the efficiency of government-to-person payments; and 5) Promote water, sanitation and hygiene, including social marketing of latrines. All of these grants – other than the private sector curative care grant – focused on supporting government functionaries and platforms through technical assistance. In addition, other partners were engaged to provide technical support to *Ananya*’s promotion of nutrition (Emory University), newborn care (Saving Newborn Lives, Save the Children US), maternal care (Addressing Maternal Death and Disability, Columbia University), family planning (DKT Janani) and private sector partners (Abt Associates).

Through collaborative partnership among the grantees, BMGF and the GoB, the *Ananya* program pilot phase was substantial (**Table 1**), reaching a population of about 28 million people in the eight focus districts (**Figure 1**). Across all grants, *Ananya* sought to support the GoB to address both supply- and demand-side barriers to the implementation of eight key technical interventions by working through the MoHFW and the Integrated Child Development Services (ICDS) programs. Key interventions included: 1) Skilled attendance at birth, meeting basic public health standards for quality of care and delivery of BEmONC; 2) Safe delivery, including clean delivery, birth preparedness and a care-seeking plan in case of complications and/or emergency with emphasis on prevention and management of birth asphyxia and neonatal sepsis; 3) Preventive postnatal care services and practices for newborns and mothers, including skin-to-skin care/kangaroo mother care, clean cord care, immediate breastfeeding, early postnatal follow-up of the mother and child, with emphasis on care for low birthweight infants; 4) Exclusive breastfeeding of infants during the first six months of life; 5) age-appropriate complementary feeding for children 6-23 months of age; 6) Compliance with recommended immunisation schedules for children up to age one year; 7) Handwashing at critical times; and 8) Postpartum family planning among women who want to space and limit future births. These interventions had been included in the MoHFW and ICDS implementation plans, but the coverage and quality of these interventions were seen as poor and inadequate to produce the desired health impacts [38,39].

**Table 1:** *Ananya* interventions

Intervention platform	Grantee	Intervention description	Target population
**Household-level interventions**			
Mobile *Kunji*	SDP − BBC	Health communication job aids and stage specific messages	Antenatal and postnatal women
Television advertisements	SDP − BBC	Birth spacing and birth preparedness	Antenatal and postnatal women
Birth preparedness cards	SDP − BBC	Birth preparedness communication	Antenatal women
Mobile Academy	SDP − BBC	Phone-based training and education modules	Frontline workers and families
*Kilkari*	SDP − BBC	Phone reminder service for stage-specific messages	Antenatal women
Communication training	SDP − BBC	Skills development for interpersonal communication	Frontline workers
Job aid kits	IFHI − CARE	Reference materials used by FLWs for counseling	Frontline workers and families
Enumeration and mapping	IFHI − CARE	Ensuring access to health services from FLWs	Families
Name based tracking	IFHI − CARE	Tracking household visits by FLWs	Women and children
Antenatal home visits	IFHI − CARE	Birth preparedness and anticipatory guidance	Antenatal women
Home visit planners	IFHI − CARE	Provided to FLWs to systematically plan home visits	Frontline workers and families
IFA tablet distribution	IFHI − CARE	Birth defect prevention	Antenatal women
Referral for complications	IFHI − CARE	Complication management	Antenatal and postnatal women
ICT services for care continuum	IFHI − CARE	Increase coverage and quality of FLW services	Frontline workers and women
Breastfeeding guidance	IFHI − CARE	Breastfeeding education modules	Antenatal and postnatal women
Family planning counseling	IFHI − CARE	Expand family planning services to facilities	Postpartum women
Family planning products	IFHI − CARE	Expand family planning services to private practice	women
Family planning service reimbursement	IFHI − CARE	Expand access to family planning	Women and providers
**Community-level interventions**			
*Gupshup Potli *	SDP − BBC	Audio-based educational modules played at VHSNDs	Frontline workers and women
Interpersonal communication tools	SDP − BBC	Promote IFA, diarrhea management and family planning	Frontline workers and women
*Khirki Mehendiwali*	SDP − BBC	Radio messages about MNCH Issues	Women
Street theatre	SDP − BBC	Birth preparedness, feeding and other maternal, newborn and child health issues	Women
Mapping catchment areas	IFHI − CARE	Mapping areas covered by FLWs	Frontline workers and families
Survey registers	IFHI − CARE	Demographic records of service area population	Frontline workers and families
Subcenter platform meetings	IFHI − CARE	Training and coordination of FLWs	Frontline workers
System debottlenecking	IFHI − CARE	Process improvement for poor performing districts	Systems
SHG+Health	CM − PCI	Layering of health education into women’s groups	Women
*Saheli* trainers	CM − PCI	FLW trainers for communication and health education	Frontline workers
**Facility-level interventions**			
Facility quality improvement teams	IFHI − CARE	Management of supply chain/equip/infrastructure/human resources	Providers and facility staff
Complication review	IFHI − CARE	Improve management of and referrals for complications	Providers and facility staff
Supply chain evaluation	IFHI − CARE	Ensure appropriate supply chain pipeline	Providers and facility staff
Sanitation tools	IFHI − CARE	Products needed (e.g. gloves, soap) for sanitary behaviors	Providers and facility staff
Surveillance mechanisms	IFHI − CARE	Ongoing monitoring for quality improvement	Providers and facility staff
Referral tracking	IFHI − CARE	Mechanism to track adherence to referral procedures	Providers and postnatal women
PPH management	IFHI − CARE	Training and tools for improved treatment of PPH	Providers and postnatal women
Intravenous antibiotics for infection	IFHI − CARE	Common antimicrobials stocked for neonatal sepsis	Neonates
Antiepileptics for eclampsia	IFHI − CARE	Seizure prevention and management for eclampsia	Prenatal women
Drug inventory training	IFHI − CARE	Pharmacist training for inventory issues	Facility staff
Procurement manuals	IFHI − CARE	Processes for drugs and equipment management	Facility staff
Resuscitation training	IFHI − CARE	Education on neonatal resuscitation	Providers and facility staff
Extra care for preterm babies	IFHI − CARE	Education on care of babies after preterm birth	Providers and postnatal women
AMANAT B: BeMONC skills	IFHI − CARE	Training for basic emergency practices	Providers and facility staff
AMANAT V: CeMONC skills	IFHI − CARE	Training for comprehensive emergency practices	Providers and facility staff
Delivery skills training	IFHI − CARE	Complication management	Providers and facility staff
Mobile nurse training	IFHI − CARE	Skills training and practicums for nursing staff	Providers and facility staff
Nurse mentoring	IFHI − CARE	Ongoing support and education for nurses	Providers and facility staff
District dashboards	IFHI − CARE	HMIS monitoring data for district performance	Facility staff

AMANAT – *Aapatkalin Matritva evam Navjat Tatparta (*or Emergency Maternal and Neonatal Preparedness), BBC – BBC Media Action, BEmONC – Basic Emergency Obstetric and Newborn Care, CEmONC – Comprehensive Emergency Obstetric and Newborn Care, CM – community mobilisation, FLW – frontline worker, HMIS – Health Management Information System, ICT– information communication technology, IFA – iron-folic acid, IFHI – Integrated Family Health Initiative, PCI – Project Concern International, PPH – postpartum hemorrhage, SDP – Shaping Demand and Practices, SHG – self-help group, VHSND – Village Health Sanitation and Nutrition Day

#### Integrated Family Health Initiative (IFHI)

**Incremental learning approach for improving FLW interactions and functionality.** In order to achieve the ambitious goals of the IFHI grant, CARE India sought to support the GoB to implement interventions across community and facility-level delivery platforms. At the community level, they simultaneously targeted three cadres of FLWs from two programs [ANMs and ASHAs from Health and Anganwadi Workers (AWWs) from ICDS] in an Incremental Learning Approach (ILA) implemented through the health subcentre platform, which was formally created by the state government for this purpose [40]. In the pilot phase, CARE India implemented the subcenter model across all 137 blocks of the eight pilot districts, ultimately covering 2550 health subcentres and incrementally training approximately 5000 ANMs, 20 000 AWWs and 20 000 ASHAs. Typically, participants in monthly ILA sessions included 10 ASHAs, 10 AWWs, two ANMs (and after 2013, one Lady Supervisor of the ICDS program) – all the functionaries of the Health and ICDS programs of one health subcentre area who together provided services to a defined population of 8000-12 000. Each monthly session was structured to include three sequential components: 1) Review of actions taken and lessons learned since the previous month’s meeting, 2) Introduction of two new topics, and 3) A negotiated set of simple, doable new actions for the next month based on each of the two new topics. The new topics were subsets of any two of the five broad domain areas that were the priorities of MoHFW and ICDS programs: maternal health, neonatal health, child health, nutrition, and family planning. More than 30 key topics were covered over a period of 15 months in all health subcentre areas in the eight districts of Bihar under IFHI. The topics also included basic processes such as universal name-based tracking to minimise exclusion and ensure coverage of the entire population, the use of Mobile *Kunji* as a job aid for health messaging, and the use of data in the subcentre meetings to emphasise gaps in coverage of key indicators. Emphasis on key content was maintained through a structured home-visit planner that helped FLWs to schedule timely contacts as well as focus on the most age-appropriate issues at each contact. Thus, each monthly session built incrementally on previous knowledge, skills and practices and attempted to translate technical interventions into “doable” actions which could be implemented by FLWs, adopted by families, and effectively reviewed by supervisors and facilitators utilising data. Within the larger ILA implementation, mHealth tools and approaches to improve FLW motivation and teamwork were also developed and rigorously tested through randomised controlled trials, the results of which are reported elsewhere [41,42].

**Quality improvement (QI) in health facilities.** *Ananya* also sought to improve BEmONC in pubic facilities to reduce or treat perinatal complications and to ensure referral of more complex cases to higher-level care facilities, principally district-level hospitals. Quality improvement (QI) efforts at PHC facilities utilised a collaborative, team-based approach supplemented by mentored skill-building. Efforts focused on interventions to improve quality of clinical care and counseling, and interventions to address gaps in infrastructure, equipment, supplies, and infection control such as biomedical waste management, decontamination and sterilisation of equipment and linen. QI teams were formed at all facilities and were responsible for identifying and addressing administrative, reporting, and managerial gaps through routine gap analyses and the development of comprehensive actions plans [43]. CARE India helped to establish multiple tools and systems to monitor clinical improvements at facilities, including a facility self-assessment tool to assess the basic functionality and utilisation of services. CARE India also established “mini skill labs” and mobile nurse training teams which utilised mobile nurse mentors and mannequin-based simulations to provide bedside coaching to clinical staff nurses on BEmONC practices in real-life scenarios [44,45]. These programs were launched in 80 facilities in the initial eight focus districts, with subsequent scale-up through the *Aapatkalin Matritva evam Navjat Tatparta (*AMANAT, or Emergency Maternal and Neonatal Preparedness) program (see below) [46].

#### Shaping demand and practices (SDP)

Complementing supply-side investments through CARE India in FLWs and facilities, *Ananya* also sought to increase demand for and adoption of priority health behaviours at the community level through its SDP grant to BBC Media Action. They used human-centered design methodology to develop and implement a “360-degree” communication strategy which included multiple complementary channels intended to saturate communities with priority RMNCHN messages [47]. BBC Media Action adopted a “media agnostic” approach, wherein the same content was designed to be adapted and delivered across multiple communications platforms, including television, radio ads, mobile telephones, and outreach activities such as street theatre and community groups. Multiple advertising campaigns were launched, with content focused on birth spacing, birth preparedness, and complementary feeding [48]. Complementing parallel efforts by other grantees to improve the outreach of FLWs, a significant focus of BBC Media Action was on improving the quality of engagement between FLWs and families by refreshing their knowledge of RMNCHN behaviours and improving their interpersonal communication skills. In the eight initial focus districts, BBC Media Action trained more than 40 000 ASHAs and AWWs in interpersonal communication skills and the use of behaviour change communications tools. FLWs were equipped with a suite of mHealth behaviour change communication tools, including Mobile Academy and Mobile *Kunji*. Mobile Academy is a training course for FLWs delivered via mobile phones that models effective communication along with refreshing knowledge of healthy behaviours. Mobile *Kunji* is a mobile phone-powered audio-visual job aid for FLWs designed to improve the quality of engagement with families during home visits by enhancing the credibility of FLWs among families, and by increasing FLWs’ confidence [49]. *GupShup Potli* delivered audio content via the FLWs’ mobile phones at Village Health, Sanitation and Nutrition Days (VHSNDs) and was designed to generate group discussions around healthy behaviours. *Kilkari*, a subscription-based mobile service, was also developed to reinforce RMNCHN messages by delivering stage-specific audio content directly to families’ mobile phones during pregnancy and until their child was one-year of age.

#### Women’s self-help groups (SHGs)

The BMGF also recognised that households and communities – beyond being recipients of health care services – could become producers of good health through key behaviour changes and adoption of improved practices, and by holding public and private health providers accountable for the provision of quality services. Moreover, there was disportionate burden of disease among the approximately 25% of the population that was Scheduled Castes, Scheduled Tribes and Pasmunda Muslims, who were marginalised and had low coverage of interventions. These communities needed to be mobilised to increase their individual and collective agency to demand and utilise RMNCHN and sanitation services and take household- and community-level actions. In 2011, the BMGF funded *Parivartan* (“Transformation” in Hindi), implemented by PCI, with the strategic objective of influencing RMNCHN and sanitation behaviours among women of reproductive age from the most marginalised communities in the eight focus districts [50]. *Parivartan*’s social behaviour change strategy included Participatory Learning and Action, social mobilisation and empowerment, and SBCC interventions designed to improve RMNCHN/sanitation-related knowledge and practices, and ultimately to improve health-seeking behaviours and to complement the efforts of CARE India and BBC Media Action. Within the traditional SHG model, they integrated 11 RMNCHN and sanitation modules addressing antenatal care, birth preparedness, postpartum and newborn care, exclusive breastfeeding, complementary feeding, hygiene and sanitation. At the collective level, the groups were designed to engender social cohesion and foster collective action. At the individual level, women were taught critical RMNCHN and sanitation messages and empowered to develop their confidence, self-esteem, and self-agency. As a result, these “health-layered” SHGs provided marginalised women with a more powerful voice and a platform to advocate for improvements in the quality of health services and to influence key RMNCHN and sanitation behaviours in their households and communities. Village volunteers – mostly young women – from the community with basic education and leadership and communications skills (called *Sahelis*) facilitated the practice of healthy behaviours through regular RMNCHN and sanitation sessions at SHG meetings, participatory game-based delivery of behaviour change modules, and complementary activities such as accompanying women to antenatal and postnatal care visits. In this initial phase of SHG implementation starting in late 2011, *Parivartan* worked with approximately 26 000 health-layered SHGs across the eight focus districts, including 18 000 groups formed by PCI and 8000 GoB-run SHGs through the JEEViKA program.

### Preparation for scale-up (2013)

All the above ‘solutions/innovations’ which constituted the core package of the *Ananya* program were untested at the scale of operations envisaged, even in the eight focus districts. This necessitated rigorous piloting, facilitation and implementation support, and monitoring and concurrent evaluation to establish proof-of-concept and to appropriately refine interventions for eventual scale-up statewide and produce globally relevant learning.

The BMGF and partners worked with the GoB to identify a common minimum package of interventions from *Ananya* as well as other development partners to be scaled up across Bihar [51]. This package included investments in the health subcentre platform, digital mHealth communication tools including inter-personal communication tools for FLWs, and quality improvement at health facilities, including nursing skills laboratories and mobile nurse trainers for mentoring. The expressed intention was to adapt innovative solutions from the pilot phase for larger scale, design operating manuals for government functionaries’ understanding and use, and transfer ownership and supervision of the innovations fully to the government for scale-up across the state by leveraging central government funding and resources for implementation.

As a next step in this process, an approach called “twinning” was conceived early in 2013 and implemented mostly in the second half of that year. This was a strategy to rapidly test the scalability of the innovations through significantly less external resources and the readiness of government systems in non-pilot districts to adopt and sustain the selected interventions that had been piloted in the eight focus districts. Each of the original eight CARE India district teams identified one neighbouring district in which to rapidly implement “light touch” versions of the ILA and QI interventions, which meant that programs would be catalysed by teams of BMGF partners that were smaller than in the original eight districts. These small teams were assigned to the new districts and were supervised by experienced district managers and block coordinators from the focus districts. The lower intensity program model also included fewer subcentre-level facilitators and fewer topics in the ILA modules. In addition, and in keeping with a lower-intensity program model, BBC Media Action trained government departments and officials as master trainers, who in turn trained FLWs in the use of their inter-personal communication tools – Mobile *Kunji* and Mobile Academy – which were both rolled out in parallel with the ILA and QI interventions. Around this time, PCI began engagement with the JEEViKA program to transfer to them the financial and program management of *Parivartan* SHGs as well as to scale up the model for health layering upon JEEViKA SHGs in collaboration with the World Bank as key funder of the JEEViKA program. Overall, there was an implicit assumption that the GoB had systems readiness and necessary technical and program expertise in taking successful solutions to scale. The governance mechanisms under the partnership, especially the partnership coordination cell and program advisory committee meetings, ensured that critical conversations took place among the GoB decision makers to enable scale-up.

### Bihar program scale-up (2014-2017)

#### Statewide Bihar Technical Support Program

Multiple converging factors led to a significant shift in the *Ananya* model in 2014. The original *Ananya* design included a pilot phase, which would inform both the what (ie, innovative tools and processes) and the how of expansion of technical assistance statewide in the latter phase of the first five-year grant. Budgetary resources available per implementation unit for deploying implementation facilitators at the scale of 38 districts were to be significantly smaller than was available during the pilot phase. This necessarily meant that facilitation approaches would need to be more resource efficient, such as technically more intensive state-level support but with reduced human resources to facilitate field operations at block and district levels.

Despite successes in the pilot phase [41,42,50,51] and effective advocacy by all partners with the government to adopt, apply and scale up successful solutions from the pilot phase, the effectiveness and fidelity of scaling-up by the GoB were severely limited by crippling and chronic health system bottlenecks and breakdowns across key “building blocks” of the health system, such as limitations in the procurement and supply chain of critical commodities, as well as the lack of human resources, infrastructure, timely funds flows and information systems. The SWASTH program also had a fixed end-point in 2016, with limited space to stretch beyond delivering mutually agreed inputs and testing them for robustness and sustainability. The GoB launched their own Human Development Mission (*Mission Manav Vikas*), covering a broad range of social sector issues and responding to the 12^th^ Planning Commission (2012-2017), creating a roadmap for improving key human development indicators [52]. In 2013, the Government of India also launched the RMNCH^+^A initiative, and negotiated with different development partners to streamline technical support to states [39]. Several implementation-support partners were working in Bihar, and there was desire on the part of the GoB to coordinate the activities of the various partners in Bihar. BMGF was chosen the lead partner for Uttar Pradesh and Bihar, and other donor partners were chosen to lead in other states. The approach was for partners to establish State-level RMNCH^+^A Units (SRUs), or “think tanks” in each state, and a National RMNCH^+^A Unit (NRU) in Delhi. Each of these bodies would be constituted by all partners supporting the state in these domains, and the lead partner was to play the coordinating role among partners. As lead partner in Bihar, BMGF was responsible for strengthening the public health system to facilitate coordinated action among development partners in ten high priority districts. BMGF proposed that innovative approaches from each partner be brought together into a commonly owned package to be offerred to the GoB as a menu of options for implementation statewide, after further testing as necessary. It was proposed that this group could guide and support the GoB in implementation using technical assistance mechanisms suited to their individual team structures.

To improve health outcomes at scale, in addition to increasing the coverage and improving the quality of critical PHC interventions, there was a recognition that the *Ananya* partners’ approach also needed to focus on helping the GoB to strengthen the performance management of the Bihar health system, while supplementing the work in progress in rebuilding health system building blocks. Accordingly, the BMGF expanded their scope to invest in technical assistance to develop the leadership and management capabilities of key government leaders, and address specific system and policy-level barriers in human resource management, essential drug and equipment procurement and supply chain management, health information management systems, management of private sector engagement in public health programs, and financial planning and expenditure management.

In late 2013, a model of technomanagerial support to the GoB’s health and ICDS programs was developed – the Bihar Technical Support Program (BTSP) (**Table 2**) – incorporating lessons from *Ananya* as well as the twinning phase, and introduced to respond to government requests for assistance. A SRU was formed in late 2013, comprised of full-time technical experts from CARE India in support of the GoB, a dedicated communications expert from BBC Media Action, and deputed technical experts from other development partners, including child health from UNICEF and NIPI, maternal health from DFID, and adolescent health from UNFPA. By mid 2014, district teams comprised of three heads focusing on health systems, public facilities and outreach functions, and sub-district/block coordinators (one per block across the state instead of four per block in the focus districts during the pilot phase) were in place through CARE India. The complementary Concurrent Measurement and Learning team, with block, district and state Monitoring, Learning and Evaluation officers was also established statewide. Importantly, however, while government ownership allowed for scale-up across the state, the intensity of human resources for implementation and facilitation support to GoB functionaries was reduced by an estimated 75% from the eight-district pilot phase at the block level.

**Table 2:** Bihar Technical Support Program strategic objectives

Intervention	Strategic objective	Description
**Facilities:**		
**Quality improvement**	Implementation of quality improvement (QI) teams to incrementally drive facility-based changes	▪ Infrastructure: Basic equipment (eg, delivery tables, blood pressure cuffs, scales)
		▪ Supplies: Medications and sanitation tools (eg, gloves, basic drugs)
		▪ Documentation and record keeping
		▪ Implementation of basic clinical care practices: infection control, basic care of newborns and mothers (ie vital signs, skin-to-skin care, exclusive breastfeeding, warning sign identification)
		▪ Management: Utilisation of dashboards, checklists, complication review, and referral tracking
**Capacity building**	Nurse mentoring and training for facility staff	▪ Skills laboratories, mentoring and on-job training for management of complications, stabilisation referrals, documentation and tracking
**Communities:**		
**FLW perfromance improvement**	Capacity building for frontline workers (FLWs)	▪ Health subcenter platform meetings: planning, reviews, coordination, incremental capacity building for FLWs
		▪ Name-based tracking: System to ensure that key health services reach all households
		▪ Job aid tools: Empower FLWs to plan and schedule household visits
		▪ Surveillance systems: Track serious health events and facilitate referrals and care
**Health systems:**		
**Addressing systems-level constraints**	“Debottlenecking” systems and addressing critical gaps in the Bihar health system	▪ Hiring of specialist providers for Comprehensive Emergency Obstetric and Newborn Care facilities (eg, obstetricians, anesthetists, pediatricians)
		▪ Appointed technical officers to State Health Society to oversee care verticals (eg, Maternal, Newborn and Child Health, Reproductive Health)
		▪ Developed comprehensive Human Resources policy for doctors and nurses
		▪ Rolled out 42-point facility inspection system
		▪ Provided comprehensive procurement manual to establish procedures for all stages of procurement
		▪ Information technology-enabled inventory management system rolled out
		▪ Provided detailed gap analysis of blood banks
**Policy:**		
**Planning & policy guidance**	Provision of planning and policy technical support to Government of Bihar officials at block, district, and state levels	▪ Professionalise contracting of private providers
		▪ Improve district and block annual plans based on gap analysis
		▪ Strengthen state-level planning and funds-release capacity
		▪ Strengthen government-to-person payment systems
**Cross-cutting:**		
**Leadership & management development**	Capacity-building and skills development through increased data utilization and focus on health outcomes	▪ Develop a focus on health outcomes, rather than processes
		▪ Create a shared mental model with a clearly-articulated mission communicated across the public health system
		▪ Integrate key messages into the government planning processes
		▪ Strengthen internal data processes (eg, Health Management Information Systems) and utilisation of technology-based platforms for tracking and analysis
		▪ Develop “concurrent monitoring systems” using dashboards for program performance monitoring
		▪ Support supervisory structures for FLW and facility staff


With support of the BTSP, on the health systems side, the program shifted its focus toward system strengthening and developing program leadership capabilities in the Department of Health and Family Welfare and the ICDS program under the Department of Social Welfare in Bihar. Thus, the BTSP sought to coordinate with both the Departments and Ministries within the state government. The effectiveness of the BTSP was predicated on persuading existing government officials and programs to take ownership of and drive key programs and service delivery platforms to improve health and nutrition outcomes across the state. Building on the first phase of *Ananya* in the eight focus districts, the scope and mandate of statewide scale-up by the GoB with BTSP technical support was ambitious. Multiple technical domains were to be strengthened across around 600 public sector hospitals and 200 000 FLWs statewide to provide critical RNMCHN services to more than 10 million mothers and their babies by 2017. To achieve these objectives, the BTSP sharpened its focus to prioritise four strategic areas: 1) Quality of care at facilities, including both BEmONC and comprehensive emergency obstetric and newborn care (CEmONC) facilities (district and referral hospitals); 2) Strengthening home/community-based health and nutrition interventions; 3) Health and nutrition system-level improvements to sustain the enabling environment that would accelerate RMNCHN interventions; and 4) Policy guidance to the GoB (**Table 2**).

#### Quality of care at facilities: Mobile Nurse Mentoring Program (AMANAT)

By mid-2014, the Mobile Nurse Mentoring Program had covered 80 of the 137 PHC facilities in the eight-district pilot phase [43]. Learning from this phase informed the design of the mentoring program that began in early 2015 in the scale-up phase, called AMANAT [46]. The basic structure of the nurse mentoring program in the scale-up phase remained the same as in the pilot phase, consisting of a pair of qualified and specially trained nurse mentors travelling out daily to PHCs from a base in the district town, providing onsite and bedside mentoring to 5-8 staff nurses in each PHC for a period of about 50 days spread over 6-8 months. Each pair of nurse mentors covered four PHCs in parallel over this period, and besides providing bedside coaching on live cases, they administered a set of incremental modules covering perinatal care and family planning designed to cover most BEmONC signal functions. However, the structure, style and content of the next phase of the clinical mentoring program were refined. The clinical mentoring program also included realistic simulations of maternal and neonatal complications (such as newborn asphyxia and maternal postpartum hemorrhage) introduced by the University of California-San Francisco (UCSF) and Pronto International [44,45,53]. By early 2017, when the AMANAT mentoring ended, it had covered 320 of the highest delivery volume government facilities in addition to the 80 covered in the pilot phase, providing mentoring exposure to around 3500 staff nurses.

Simultaneously, a doctors’ mentoring program was piloted over 2014-2015 in five of the eight pilot districts, targeting support primarily for specialist doctors who were expected to provide emergency response, surgery and anaethesia services for obstetric and perinatal care in District Hospitals. Since sufficient numbers of specialist doctor mentors could not be found, the doctor mentoring component of AMANAT remained much reduced in scope compared to the nurse mentoring component in the scale-up phase.

QI was scaled-up statewide starting in early 2015, and reached 56 district hospitals and all the remaining block-level PHC facilities across the state – about 600 in total. Initially, focus was directed to improving fundamental health system building blocks (eg, infrastructure, equipment, supplies, basic infection control), with a gradual shift to improving skills through clinical mentoring. Clinical case reviews were used with increasing intensity across the state as a means to transfer ownership to facility staff to ensure quality of clinical care.

Sweeping QI efforts, including the mobile nurse mentoring program, were closely supported by robust improvements in facility infrastructure, equipment, supplies, and management [46]. Undergirding all of these efforts, CARE India sought to strengthen components of the Bihar health system, such as the deployment of critical human resources (with a focus on specialist doctors and nurses), training and supervision, data-driven reviews at multiple levels, supply chain management, and management information systems.

#### Layering of RMNCHN into JEEViKA SHGs

The Bihar Rural Livelihoods Promotion Society under the GoB and with the support of the World Bank Group, had launched the second phase of support to the JEEViKA program in 2015. The aim was to improve the livelihoods and economic security of women across Bihar through SHGs and their clustered village organisations and the federated organisations at the block level (called cluster federations), with the ambition of reaching one million SHGs in rural Bihar and 70% of the total rural population by 2022. To date, the JEEViKA program has formed more than 850 000 SHGs across the state of Bihar, reaching more than 9 million women.

The BMGF and PCI saw an opportunity to scale-up health-layered SHGs across Bihar by adapting and layering the *Parivartan* model into JEEViKA operations [54,55]. In 2015, the BMGF funded the JEEViKA Technical Support Program (JTSP) led by PCI. Although the JEEViKA program was not health focused at that time, JTSP partnered with the JEEViKA program to create and institutionalise the focus on RMNCHN and sanitation behaviours and in 2015 the $415M program included 11% allocation to RMNCHN. The JTSP led the layering of RMNCHN plus sanitation interventions upon SHGs formed by JEEViKA in 101 blocks across 11 districts, thus leading to a second stage of statewide scale-up beyond the pilot districts. Similar to the BTSP structure, the JTSP program provided technomanagerial support to the GoB’s JEEViKA program at the block and state levels to more effectively manage the JEEViKA scale-up, which included prototyping selected interventions which were subsequently scaled-up via JEEViKA SHGs. The JTSP also incorporated a multi-pronged communication initiative and strategic guidance to the GoB.

## EVALUATIONS

**Figure 3:**
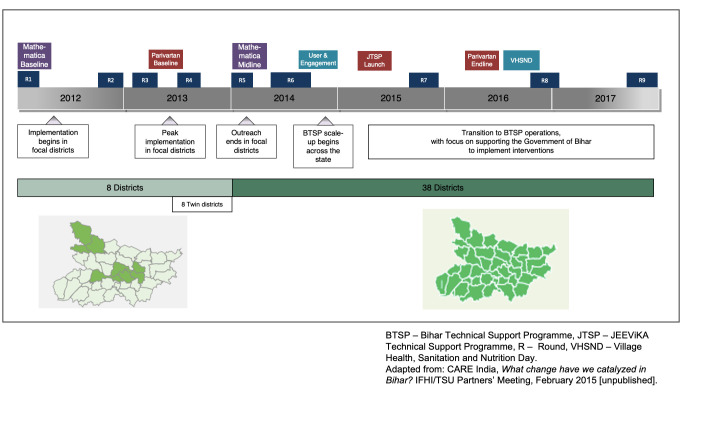
Overview of the timeline for implementation and evaluations of the *Ananya* pilot and scale-up program. GoB – Government of Bihar, JTSP – JEEViKA Technical Support Program, TSU – Technical Support Unit, VHSND – Village Health, Sanitation and Nutrition Day, BTSP – Bihar Technical Support Program. Adapted from: CARE India, *What change have we catalyzed in Bihar?* IFHI/TSU Partners’ Meeting, February 2015 (unpublished).

Key to the original design of the *Ananya* program was evaluation and analysis of the interventions being implemented (**Figure 3**). From the outset, concurrent monitoring, learning and evaluation grants were made in order to assess the progress of programs and support decision-making by the BMGF, grantees and the GoB. The following is a brief description of these data sources and evaluations (Table S3 in the **Online Supplementary Document**); further details are provided in the papers of this collection that follow.

### Difference-in-difference evaluation (Mathematica)

Mathematica implemented a large-scale quasi-experimental impact evaluation of *Ananya* that included statewide household surveys at two time points during the pilot phase – January through April 2012 (“baseline”) and January through April 2014 (“midline”). The evaluation included the treatment arm comprised of the eight focus (intervention) districts, and a comparison arm that included the remaining 30 districts in Bihar where *Ananya* was not implemented in the pilot phase. Surveys were administered to FLWs (ANMs, ASHAs, AWWs) and maternal household respondents. Our analysis focused on maternal household respondents. Surveys were conducted by an independent contractor (Sambodhi) in collaboration with Mathematica. Surveyors collected data from households selected using a multistage sampling approach which is described in detail in a subsequent paper [56]. Surveys focused on children born in the previous 12 months (ages 0–11 months) because interventions were targeted most intensively on delivery and health-related behaviours in the first year after delivery. The 2014 survey also included an additional sample of children ages 12–23 months in the sampled villages, which was not included at baseline, but which allowed the evaluation to assess additional, longer-term indicators of immunisation, undernutrition, and family planning. Mathematica used all non-focus (other) districts (n = 30) as the primary comparison group. Comparing focus and non-focus districts across both the 2012 and 2014 surveys enabled estimation of difference-in-difference as a reflection of the contribution of *Ananya* to changes in indicators over the survey period. The relative merits of this design are that it was conducted independently, used a population-weighted quasi-experimental evaluation design, examined dozens of RMNCHN indicators after two years of implementation compared to baseline data, and took secular changes in indicators into account. Once the Bihar program was scaled up statewide, the comparison group design of the pilot phase could not be maintained. Additional grants were made to Public Health Foundation of India to measure neonatal mortality statewide and a systems strengthening assessment grant was made to Oxford Policy Management. Results from these evaluations are reported separately by those organisations.

### Community-based household surveys (CHS, CARE India)

Throughout the implementation of the program (ie, during the *Ananya* pilot phase as well as the BTSP-supported scale-up phase), CARE India collected extensive survey data, including nine “rounds” from 2012-2017 which are analysed in this paper collection, and continue to the present. The CHS surveys were originally designed as an internal monitoring tool for CARE India and government staff to provide robust state and district-level estimates for a range of indicators of RMNCHN service coverage, quality, utilisation and beneficiary practices at the household level as well as to assess individual block-level performance (as pass/fail). However, over time as their use and interest of stakeholders grew over the first couple of rounds, methodological and implementation rigor was strengthened to maximise reliability. Round 1 was a pilot survey to establish the methodology and thus is not used in our analyses. Rounds 2-9 of the survey were carried out between 2012 and December 2017 (**Figure 3**). Rounds 2-5 correspond to the *Ananya* pilot phase of intensive innovation development and technical support to implementation in the focal districts (2012-13) and data were collected from the eight focus districts only, and were administered by an internal CARE India data collection team. The subsequent rounds 6-9 (2014-2017) covered all 534 blocks of the 38 districts of the state, collected by CARE India’s Concurrent Measurement and Learning unit working independently of the implementation unit to generate robust measurements. The surveys captured data for respondents in the families of children from four age groups (0-2, 3-5, 6-8 and 9-11 months) and six key intervention fields: antenatal care and birth preparedness, delivery care, postnatal care, infant feeding practices, immunisation, and family planning. A fifth age group was included (12-23 months) in rounds 6-9 in order to assess immunisation status. The age groups were selected to capture the most recent values of different indicators and thus provide non-overlapping cross-sectional samples to measure progress efficiently over closely spaced survey rounds to reduce recall bias, and to provide estimates of the most recent period. The latter was particularly important since it provided programmatically actionable values. The household surveys used a two-stage proportional random sampling with a systematic component (random start) at the individual level to generate robust district-level point estimates and state-level change in estimates for all indicators; further detail is provided in subsequent papers in this collection [54,55,57]. To assess block-level program performance (ROC*curve-based Pass/Fail), a design similar to the Lot Quality Assurance Sampling (LQAS) was used [58]. Using the random samples of respondents in small numbers from every block permitted the application of LQAS tables to provide ‘pass/fail’ measures of performance of various indicators at the block level. When aggregated, these provide point estimates at district and project or state levels. The survey instruments were designed to capture the closest approximation to global standard RMNCHN coverage indicators (subject to limited denominators from the sampled age groups), but also included a substantial number of indicators specific to the intervention processes as implemented, and designed to measure progress on the components of the Theory of Change of the program. We also assessed variation in geospatial trends in indicators over time at the block level [59].

A chief advantage of CARE India’s CHS is that the surveys spanned the entire intervention period from baseline through the scale-up period. Use of sampling techniques that approximated simple random sampling, close attention to logistical planning and continuity of survey teams of field enumerators increased efficiency and reduced costs compared with conventional cluster sampling surveys and enabled examination of an expansive number of RMNCHN indicators (300+). There was no comparison group, however, as sampling during the pilot phase was restricted to the eight pilot districts, and during rounds 6-9 implementation was statewide. Thus, evaluation of these data focused on changes over time, which was the primary purpose of these survey, but could not take secular trends into account.

### Facility-level assessments

Several facility-based assessments were implemented and/or facilitated by CARE India. A facility web-based routine monitoring system was developed, using data generated by visiting nurse mentors to compare reliable trends of maternal and neonatal complications across geographies and time. Direct Observation of Deliveries (DOD) was conducted by trained nurses who assessed providers' skills and quality of care offered in BEmONC and CEmONC facilities; these data were used to assess the effectiveness of the nurse mentoring program in improving the quality of intrapartum care, particularly for cases of normal delivery and prevention of complications. Provider Knowledge Assessments (paper-based tests) were used to assess knowledge of nurse mentoring mentees in BEmONC and CEmONC facilities, and Comprehensive Facility Assessments (CFAs) were conducted to identify gaps in health facility readiness for service delivery, including interviews with staff on key practices and direct observation of infrastructure, functionality of equipment, drugs and consumables. Since 2016 all these assessments have moved to digital data capture platforms.

### Assessment of quality improvements

Johns Hopkins University implemented a comprehensive analysis and description of the QI approach and an assessment of its success covering the period of 2014-2017, drawing on multiple facility-level assessments (above) including the DOD and CFA [43,46].

### Nurse training simulations and team training assessments

PRONTO International’s simulation and team-training in primary care was incorporated into the large-scale AMANAT nurse-mentoring program to improve intrapartum and newborn health outcomes. The intervention and data collection (further triangulated with CARE India’s AMANAT assessment data) were conducted between May 2015 and January 2017. The PRONTO components provided nontechnical and technical competencies required for team-based management of a variety of obstetric and neonatal clinical situations. UCSF assessed the effectiveness of nurse mentoring including simulations in 320 BEmONC facilities [45]. Deliveries were observed to obtain specific information on evidence-based practice indicators before and after the intervention. The two outcomes – intrapartum and newborn care composite scores – were calculated using evidence-based practice indicators. A web-based routine monitoring system developed by CARE provided data on training intensity, and simulation and teamwork-communication activities.

### Evaluation of communications interventions

In order to evaluate the impact of the BBC Media Action interventions, the CHS and Mathematica data described above were used to compare women exposed to Mobile *Kunji* vs those unexposed to the tool in order to assess the differences in their health-related knowledge and behaviours [47]. Furthermore, two internal evaluations implemented by BBC Media Action were analysed including the Usage and Engagement study and the VHSND survey. The Usage and Engagement study collected surveys from maternal respondents to extensively examine the impact of Mobile *Kunji* on knowledge, attitudes and practices of birth preparedness, nutrition and family planning. The VHSND survey was similarly implemented to elucidate the impact on maternal knowledge, attitudes and practices from BBC Media Action’s low-tech interpersonal communication tools and mobile-based health communication service used by FLWs. Survey data was analysed to comprehensively assess the impact of these tools on birth preparedness practices, maternal and neonatal care, complementary feeding, family planning methods and immunisation practices.

### Impact evaluation of SHGs

Population Council led the *Parivartan* evaluation to understand the impact of layering RNMCHN and sanitation into women’s SHGs shaped around livelihood, empowerment, and poverty reduction. Surveys (baseline in 2013, midline in 2014, and endline in 2016) were administered to women in *Parivartan* SHGs with health layering or in JEEViKA SHGs as a comparison, as well as women who were not in SHGs but who were exposed to other *Ananya* interventions. With the phase out of the *Parivartan* program, emphasis shifted to understanding the influence of health layering of government-led JEEViKA SHGs at statewide scale using CHS data, which included a query to women who had given birth in the last 12 months about their participation in SHGs [54,55].

**Evaluation of equity of program impact**

Program impacts were assessed for the most compared to the least marginalised women in Bihar, India [60] using an intersectional approach based on wealth and caste [61]. Differences in impacts for women at the two extremes – the least vs. the most disadvantaged – by wealth and caste were estimated using difference-in-difference estimators from Mathematica data collected at the beginning (2012) and after two years of program implementation (2014). Changes in disparities over time were also analysed using eight rounds of CHS (2012-2017). Key questions addressed were: were there disparities in indicators at baseline, and did the disparities change over the course of the program during the piloting and scale-up phases? 

## 

## LOOKING AHEAD: PAPER COLLECTION ON LEARNING FROM *ANANYA*

The *Ananya* initiative generated an expansive amount of data which had not been comprehensively analysed, synthesised and widely shared. Therefore, much of the *Ananya* story – with its invaluable experiences, insights, and evidence drawn from robust quantitative and qualitative data – had not yet been told.

To address this gap, this journal has commissioned a series of papers led by Stanford University – authored by multiple universities, evaluators, and implementing organisations – to provide a unified, comprehensive and evidence-based analysis of the successes, challenges, and insights from *Ananya* and subsequent statewide scale-up (**Table 3**). We aim to provide a deeper understanding of the dynamic interrelations between the *Ananya* program’s interventions and delivery platforms, the public health system in which it was embedded, and the communities and households that they sought to impact. We also aim to inspire additional analyses of more specific elements of *Ananya* that will further advance learning from this unique public health program. Capturing lessons from *Ananya* and the statewide scale-up of *Ananya* innovations will assist program managers and policymakers in India and across the world to more effectively design and implement RMNCHN programs, and to improve the performance of community-based, outreach and facility-based health systems.

**Table 3:** Papers in the collection in the *Journal of Global Health* on Learning from *Ananya:* Lessons for primary health care performance improvement

Paper	Objective
Viewpoint: Learning from *Ananya*: Lessons for primary health care performance improvement	This commentary provides a broad overview of key findings from *Ananya* evaluations and reflections on lessons learned for researchers, program managers and policy makers on achieving impact through primary health care programs.
1) Improving primary health care delivery in Bihar, India: Learning from piloting and statewide scale-up of *Ananya*	This paper provides a broad overview of *Ananya* and subsequent statewide adaptation and scale-up in Bihar, India, including the background and context, key objectives, interventions, delivery approaches and evaluation methods, based on review of published literature, partner and governmental documents, and qualitative interviews of partner staff.
2) Impact of the *Ananya* program on reproductive, maternal, newborn and child health and nutrition reproductive, maternal, newborn and child health and nutrition (RMNCHN) in Bihar, India: early results from a quasi-experimental study	This paper summarises changes in RMNCHN indicators during the first two years of pilot-phase implementation (2012-2013), comparing focus (n = 8) and non-focus (n = 30) districts in Bihar, India, using Mathematica data.
3) Trends in reproductive, maternal, newborn and child health and nutrition indicators during five years of piloting and scaling-up of *Ananya* interventions in Bihar, India	This paper examines trends in RMNCHN indicators in the program’s implementation districts in Bihar, India, in 2012-2017 using Community-based Household Survey (CHS) data.
4) Geospatial variations in reproductive, maternal, newborn and child health and nutrition indicators at block level in Bihar, India, during scale-up of *Ananya* program interventions	This paper examines levels and trends of a set of RMNCHN indicators at block-level during the statewide scale-up phase of the *Ananya* program in Bihar, India using CHS data in 2014-2017.
5) Impact of mHealth interventions for reproductive, maternal, newborn and child health and nutrition (RMNCHN) at scale: BBC Media Action and the *Ananya* Program in Bihar, India	This paper describes changes in RMNCHN indicators related to large-scale program implementation of innovative mHealth tools in Bihar, India, using multiple sources of survey data in 2012-2017.
6) Health impact of self-help groups scaled up statewide in Bihar, India	This paper examines RMNCHN and sanitation impacts of self-help groups (SHGs) at scale, including characteristics associated with SHG performance in Bihar, India, using CHS data from 2014-2017.
7) Health layering of self-help groups: impacts on reproductive, maternal, newborn and child health in Bihar, India	This paper examines RMNCHN and sanitation impacts of the *Parivartan* model of health-integrated SHGs, the impact of health-layered SHGs scaled up by the government, and the additional contribution of health-layering to health impact of SHGs formed initially for purposes of livelihoods and microfinance promotion, over the time period 2014-2017 in Bihar, India, using CHS data.
8) Implementation of a quality improvement initiative for reproductive, maternal, neonatal and child health and nutrition in Bihar, India	This paper provides a description, including key results, of a comprehensive, statewide quality improvement (QI) initiative to improve the quality of RMNCHN services in public facilities in Bihar, India during 2014-2017 using CARE program documents, and Comprehensive Facility Assessment, Direct Observation of Delivery, and CHS data.
9) Using a mobile nurse mentoring and training program to address a health workforce capacity crisis in Bihar, India: Impact on essential intrapartum and newborn care practices	This paper examines the impact of the AMANAT intervention on nurse-mentees’ competency to provide such services in Bihar, India during 2014-2017 using Direct Observation of Delivery and AMANAT provider knowledge and facility infection control data.
10) Simulation and team training embedded nurse mentoring program and improvement in intrapartum and newborn care in a low-resource setting in Bihar, India	This paper assesses the effectiveness of nurse-mentoring, including simulations, on intrapartum and newborn care practices in 320 basic emergency obstetric and neonatal care facilities in 2015-2017 in Bihar, India, using Direct Observation of Delivery (DOD) and Facility Information System data.
11) Evaluation of a large-scale reproductive, maternal, newborn and child health and nutrition program in Bihar, India through an equity lens	This paper compares the impacts of the large-scale RMNCHN program implemented statewide in Bihar, India among the most marginalised compared to the least marginalised women, using Mathematica (2012-2013) and CHS (2014-2017) data.
Viewpoint: Best practices in global health evaluation: Reflections on learning from an independent program analysis in Bihar, India	This commentary provides reflections on best practices for complex program evaluations for low- and middle-income countries (LMICs), with an aim to improve learning, policy and program impact in LMIC contexts.

## Additional material

Online Supplementary Document
