# Supplementary material for: Improving primary health care delivery in Bihar, India: Learning from piloting and statewide scale-up of Ananya
Source: J Glob Health. 2020 Oct 23;10(2):021001. doi: 10.7189/jogh.10.021001 (PMC7757841; doi:10.7189/jogh.10.021001)
Supplement: Online Supplementary Document [file jogh-10-021001-s001.pdf]

Supplemental Table 1a: Sample Registration System (SRS) data for India (nationwide) and Bihar (2011-2016)

**Indicator**	**Bihar (*2011)**	**Bihar (2014)**	**Bihar (2016)**	**India (2011)**	**India (2016)**
Maternal mortality ratio (MMR) (per 100,000 live births)	219	208	165	167	130 (2014-16)
Infant mortality rate (IMR) [0-1 year] (per 1000 live births)	44	42	38	44	34
Neonatal mortality rate (NMR) [0-28 days] (per 1,000 live births)	29	28	N/A	31	24
Early NMR [within 7 days of birth] (per 1,000 live births)	24	20	18	24	18
Total fertility rate (TFR)	3.6	3.2	3.3	2.4	2.3
Under-5 mortality rate (per 1,000 live births)	59	56	37	55	39

Source: Special Bulletin on Maternal Mortality in India (http://www.censusindia.gov.in/vital_statistics/SRS_Bulletins/MMR%20Bulletin-2014-16.pdf), and http://www.censusindia.gov.in/vital_statistics/SRS_Statistical_Report.html

Supplemental Table 1b: National Family Health Survey [NFHS-3 (2005/2006) and NFHS-4 (2015/2016)] data for India (nationwide) and Bihar

**Indicator**	**NFHS-3 (2005-2006) Bihar**	**NFHS-4 (2015-2016) Bihar**	**NFHS-3 (2005-2006) India**	**NFHS-4 (2015-2016) India**
Female literacy (age 15-49)	37.0%	49.6%	55.1%	68.4%
Contraception prevalence rate (CPR)	34.1%	24.1%	56.3%	53.5%
$\geq$4 antenatal care (ANC) visits	11.2%	14.4%	37.0%	51.2%
Institutional delivery rate	19.9%	63.8%	38.7%	78.9%
Fully immunised children (age 12-23 months)	32.8%	61.7%	43.5%	62.0%
Children under 6 months exclusively breast fed	28.0%	53.5%	46.4%	54.9%
Iron-folic acid (IFA) consumption 100+ days	6.3%	9.7%	15.2%	30.3%

Source: National Family Health Survey, India: http://rchiips.org/nfhs/

Supplemental Table 1c: District Level Household and Facility Survey (DLHS-3, 2007/2008) data for India (nationwide) and Bihar

**Indicator**	**Bihar**	**India**
Institutional delivery rate (%)	27.5	46.9
$\geq$3 ANC visits (%)	26.3	49.7
Mothers who received postnatal care within 2 days of delivery (%)	25.6	47.5
Mothers who consumed IFA for 100+ days during pregnancy (%)	5.6	46.9
Children under age 3 years breastfed within one hour of birth (%)	16.0	40.5
Children under age 6 months exclusively breastfed (%)	38.3	45.0
Children 12-23 months fully immunized	41.4	54.0
Modern contraceptive prevalence rate (%)	29.4	48.2

Source: http://rchiips.org/

Supplemental Table 2: Initial outcome targets for the *Ananya* programme, May 2010*

Indicator	Baseline	Source	Target (2015)	Source
Maternal mortality ratio	312	SRS 2008**	175	GoB state plan
Neonatal mortality rate	40	NFHS-3 (2005-2006)***	28	BMGF internal recommendation
Infant mortality rate	62	NFHS-3 (2005-2006)	44	GoB state plan
Under-5 mortality rate	85	NFHS-3 (2005-2006)	55	GoB state plan
% Underweight children (0-5 years)	55	NFHS-3 (2005-2006)	46	GoB state plan

*These targets were revised in subsequent project stages
** Sample Registration System (SRS) Statistical Report 2008 (source: Office of the Registrar General & Census Commissioner, India)
*** National Family Health Survey 3 (NFHS) (2005-2006) (source: International Institute for Population Sciences (IIPS) and Macro International)

Supplemental Table 3: Description of data sources used in analyses of *Ananya* pilot and Bihar statewide scale-up effects

Data source	Grantee	Location	Timeline	Data methods/sample size
Baseline/midline difference-in-differences evaluation	Mathematica	Eight districts that were part of *Ananya* pilot phase	Baseline: Jan-April 2012 Midline: Jan-April 2014	Difference-in-differences comparison group design using data collected from a cohort of beneficiaries at baseline (Jan-April 2012) and from a new cohort of beneficiaries at midline (Jan-April 2014). Compared changes in outcomes between baseline and midline for beneficiaries in the eight focus districts (where *Ananya* interventions were implemented) to the changes in outcomes for beneficiaries in the remaining 30 non-focus districts. Data were collected from a representative sample of women across Bihar who had given birth in the previous 12 months baseline: 13,069 surveys / midline: 12,015 surveys
Community-based Household Surveys	CARE India	Rounds 2-5 (Bihar focus districts) Rounds 6-9 (all 38 districts)	Annually 2011-2017	Odds ratios of RMNCHN indicators, for rounds 2-5 and 6-9 separately to identify whether an increase occurred. Separately, a comparison of round 5 and round 6 was made to evaluate the switch to the Bihar Technical Support Programme. An overall trend from rounds 2-9 was calculated to examine trends over the entire study period.<br><br><u>Round/sample size</u><br>Round 2-5: 10,408 (~2,602 per round)<br>Round 6-9: 62,685 (~15,671 per round)
Usage and Engagement (U&E) Study	BBC Media Action	8 focus districts	October-December 2014	Surveys of maternal household respondents and FLWs on knowledge, attitudes and behaviours in topic areas addressed by Mobile *Kunji*.<br><br>Sample size:<br>U&E – HH: n= 3379<br>U&E – FLW: n=582
Village Health, Sanitation and	BBC Media Action		July-September	Two separate surveys querying women exposed to *Gupshup Potli* (GSP) and Interpersonal Communication

Nutrition Day (VHSND) survey		8 focus districts	2016	(IPC) regarding their knowledge, attitudes and behaviours related to the topics discussed by these tools.<br>Sample size:<br>VHSND-FLW: n=518<br>VHSND-HHGSP: n=2608<br>VHSND-HHIPC: n=2002
Parivartan women's self-help group (SHG) evaluation	Population Council	8 focus districts	Baseline (2013)<br>Midline (2014)<br>Endline (2016)	Three surveys were undertaken: a baseline (2013 with 744 SHG groups), midline (2014 with 1026 SHG groups) and endline (2016 with 1458 SHG groups). Surveys sought to understand how *Parivartan* impacted RMNCHN outcomes in both newly formed and in traditional SHGs (formed to promote livelihoods and access to microcredit) in India. Surveys were taken from women participating in groups as well as *Sahelis* or group leaders. At the individual level, RMNCHN topics evaluated included: health, nutrition, and sanitation modules, including antenatal care, birth preparedness, postpartum and newborn care, exclusive breastfeeding, and complementary feeding. At the group level, questions about social cohesion, leadership, mobilization strength, accountability and organized collective action were asked.
Facility web-based routine monitoring system	This study assessed the effectiveness of nurse-mentoring including simulations on intrapartum and newborn care	36 districts in Bihar	Between May 2015 and January 2017	320 BEmOC facilities.<br><br>1774 deliveries were observed for intrapartum and newborn care practices, of which 942 were observed post-intervention
AMANAT: Direct Observation of Deliveries	Trained nurses observed deliveries to assess	36 districts in	Pre- and post-AMANAT training (2015-2017)	Direct Observation of Deliveries by highly trained nurses.<br><br>400 BEmONC and 16 CEmONC facilities

	providers' skills and quality of care offered in facilities	the state		
AMANAT: Provider knowledge assessments	Assess AMANAT mentees' knowledge	36 districts in Bihar	Pre- and post-AMANAT training 2015-2017)	Pre- and post-AMANAT training paper-based tests<br><br>400 BEmONC and 16 CEmONC facilities
Comprehensive Facility Assessments	Conducted by CARE India to identify gaps in health facilities' readiness for service delivery	All 38 districts in Bihar	Three rounds between 2015-2017	Direct observation of infrastructure (condition of wall, floor, roof, illumination), functionality of equipment, physical counting of drugs and consumables;<br>Interviews with staff for infection control practices and human resources information;<br>Review of records for completeness, up-to-date status and information related to case load, length of stay and bed occupancy;<br>Interviews with recently delivered patients for care and counselling provided in postnatal ward
Facility Assessment Tool	Conducted independently by all facilities in the state to identify gaps, develop plans to address gaps, and prioritise actions at facility level.	All 38 districts in Bihar	Annually, between 2014 and 2017	Program data collected in all facilities
